# Targeting IGF-IR improves neoadjuvant chemotherapy efficacy in breast cancers with low IGFBP7 expression

**DOI:** 10.1038/s41698-024-00712-9

**Published:** 2024-10-03

**Authors:** Christopher Godina, Michael N. Pollak, Helena Jernström

**Affiliations:** 1 Division of Oncology, Department of Clinical Sciences in Lund, Lund University Cancer Center/Kamprad, Lund University and Skåne University Hospital, Barngatan 4, SE-221 85 Lund, Sweden; 2 Lady Davis Institute for Medical Research, Jewish General Hospital and Department of Oncology, McGill University, Montreal, QC Canada

**Keywords:** Breast cancer, Predictive markers, Cancer microenvironment

## Abstract

There has been a long-standing interest in targeting the type 1 insulin-like growth factor receptor (IGF-1R) signaling system in breast cancer due to its key role in neoplastic proliferation and survival. However, no IGF-1R targeting agent has shown substantial clinical benefit in controlled phase 3 trials, and no biomarker has been shown to have clinical utility in the prediction of benefit from an IGF-1R targeting agent. IGFBP7 is an atypical insulin-like growth factor binding protein as it has a higher affinity for the IGF-1R than IGF ligands. We report that low *IGFBP7* gene expression identifies a subset of breast cancers for which the addition of ganitumab, an anti-IGF-1R monoclonal antibody, to neoadjuvant chemotherapy, substantially improved the pathological complete response rate compared to neoadjuvant chemotherapy alone. The pCR rate in the chemotherapy plus ganitumab arm was 46.9% in patients in the lowest quartile of *IGFBP7* expression, in contrast to only 5.6% in the highest quartile. Furthermore, high *IGFBP7* expression predicted increased distant metastasis risk. If our findings are confirmed, decisions to halt the development of IGF-1R targeting drugs, which were based on disappointing results of prior trials that did not use predictive biomarkers, should be reviewed.

## Introduction

Dysregulation of type 1 insulin-like growth factor receptor (IGF-1R) signaling activates PI3K and Ras/ERK signaling pathways and leads to increased breast cancer cell growth, proliferation, and survival^1–5^. IGF-1R is closely related to its homolog, the insulin receptor (INSR), and it is common for breast cancer cells to display IGF-1R, INSR, and hybrid receptors^6–8^. Many agents have been developed to target IGF-1R^9^. To date, no phase 3 clinical trials have demonstrated a substantial clinical benefit of adding IGF-1R targeting agents to existing treatments^9^. A possible explanation is that response to chemotherapy is enhanced by IGF-IR targeting only in a subset of tumors, following the well-known precedent that adding EGFR inhibitors to chemotherapy in lung cancer is only helpful in tumors with *EGFR* mutations^10,11^. However, no predictive biomarkers for IGF-IR targeting agents have been identified. Since ~30% of breast cancer patients eventually relapse despite receiving optimal adjuvant or neoadjuvant treatment according to clinical guidelines^12^, it is crucial to rigorously evaluate novel candidate treatment strategies and to identify any subsets of patients who benefit.

**Fig. 1:**
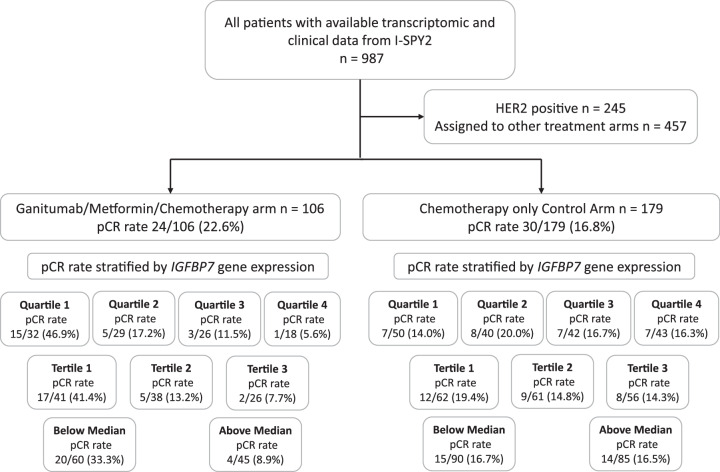
Flowchart of included and excluded patients in I-SPY2. pCR rates in the ganitumab/metformin plus chemotherapy arm and chemotherapy-alone control arm, according to *IGFBP7* expression categories.

Recently, the Investigation of Serial Studies to Predict Your Therapeutic Response With Imaging And moLecular Analysis 2 (I-SPY2) trial showed a small increase in pathological complete response (pCR) rate when the anti-IGF-1R monoclonal antibody ganitumab (AMG479), given with metformin to reduced treatment-induced hyperglycemia, was added to chemotherapy, but the effect size failed to reach the prespecified threshold for a positive result^13^. The trial design is summarized in Fig. 1. The subgroup-specific predictive Bayesian probabilities of success were all below 51%, well under the predefined threshold of 85% probability of success in a subgroup-specific, hypothetical 300-patient, 1:1 phase 3 trial^13^. Putative IGF-1R signaling axis predictive biomarkers were tested, consisting of *IGF1*, *IGF2*, *IGF-1R*, *IGFBP2*, *IGFBP3*, *IGFBP4*, *IGFBP5, INSR, IRS1, IRS2, CDH1* gene expression, IGFBP5/IGFBP4 ratio, the IGF-1 ligand score^14^, and the Creighton IGF-1R signature^15^. None was predictive of ganitumab benefit^13^.

I-SPY2 has been able to identify treatment predictive biomarkers for emerging treatments due to its unique design^16–19^ and to evaluate the efficacy of new treatments^20–23^. To further this goal, the I-SPY2-990 Data Resource has been made publicly available^20^.

It has previously been shown^24^ that unlike other insulin-like growth factor binding proteins (IGFBPs), IGFBP7 can bind to the IGF-1R, but the in vivo consequences of the IGFBP7 IGF-1R interaction are unclear. There are also gaps in knowledge concerning complex interactions between IGFBP7 and IGF-1R in the presence of anti-IGF-1R antibodies such as ganitumab. IGFBP7 binds IGF-1 and the IGF-1R in a mutually exclusive manner^24^. IGFBP7 can also bind to IGF-2^25^. Compared to other IGFBPs, IGFBP7 binds insulin with higher affinity than IGFs^24,26^. The binding of IGFBP7 decreases activation and internalization of IGF-1R in response to IGF-1/2 but at the same time sensitizes IGF-1R to insulin stimulation^24,26^. IGFBP7 was shown to prolong the surface retention of the IGF-1R under insulin/IGF-1 stimulation resulting in prolonged IGF-1R signaling in leukemia^26^. It also has been shown that IGFBP7 promotes the persistence of IGF-1R at the cell surface, prolonging insulin/IGF stimulation and enhancing Akt activation leading to mitogenic and pro-survival effects^26,27^.

Although we have presented prior evidence that high IGFBP7 expression by breast cancer tissue or high circulating levels of this protein are related to poor prognosis^28,29^, this protein has not previously been studied in relation to the efficacy of IGF-1R targeted therapies. We previously reported that the prognostic value of circulating IGFBP7 was only seen for patients who had cancers with positive IGF-1R membrane status^29^, which suggested that the interplay between IGFBP7 and IGF-1R merits further investigation. The primary aim of this study was to investigate *IGFBP7* expression as a biomarker predictive of ganitumab benefit in the neoadjuvant setting. A secondary aim was to investigate IGFBP7 as a prognostic biomarker.

## Results

### *IGFBP7* gene expression in relation to pCR rate by trial arm

The pCR rates in the patient treatment arms of I-SPY2 and in different subgroups thereof based upon *IGFBP7* expression are shown in Fig. 1. Multivariable analysis confirmed that *IGFBP7* expression was not associated with likelihood of achieving a pCR in all patients enrolled in the I-SPY2 study, including both patients who received ganitumab and those who did not (Supplementary Table 1). Descriptive statistics for clinicopathological factors are presented in Table 1 and Supplementary Table 2.

**Table 1:** Descriptive statistics of chemotherapy-alone control arm and ganitumab plus metformin arm in relation to clinicopathological characteristics in I-SPY2

			Treatment arm	
	All patients^a^ *N* = 285	Missing	Chemotherapy-alone, *n* = 179^a^	Ganitumab + metformin, *n* = 106^a^
ER/PgR+	152 (53%)	0	94 (53%)	58 (55%)
HER2+	0 (0%)	0	0 (0%)	0 (0%)
MP2	147 (52%)	0	88 (49%)	59 (56%)
Immune+	133 (47%)	0	86 (48%)	47 (44%)
DRD+	123 (44%)	3	81 (46%)	42 (40%)
PAM50 subtype		4		
LumA	54 (19%)		30 (17%)	24 (23%)
Basal	140 (50%)		86 (49%)	54 (51%)
Her2	13 (4.6%)		9 (5.1%)	4 (3.8%)
LumB	62 (22%)		40 (23%)	22 (21%)
Normal	12 (4.3%)		10 (5.7%)	2 (1.9%)
pCR	54 (19%)	0	30 (17%)	24 (23%)
IGFBP7 quartiles		5		
Q1	82 (29%)		50 (29%)	32 (30%)
Q2	69 (25%)		40 (23%)	29 (28%)
Q3	68 (24%)		42 (24%)	26 (25%)
Q4	61 (22%)		43 (25%)	18 (17%)

Control: Paclitaxel followed by anthracyclines.

^a^*n* (%).

**Fig. 2:**
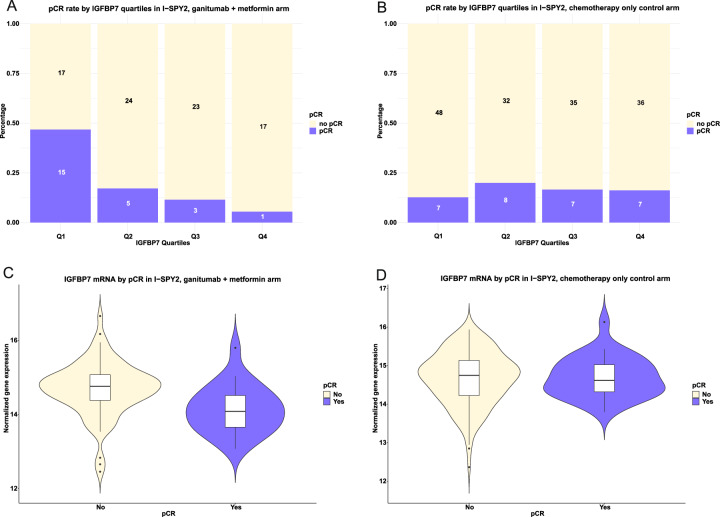
*IGFBP7* expression in relation to pCR. pCR rate by *IGFBP7* quartiles in **A** the ganitumab/metformin plus chemotherapy arm and in **B** the chemotherapy-alone control arm. The *y*-axis indicates the percentage of patients that achieved a pCR by *IGFBP7* quartiles as indicated by the *x*-axis and the raw numbers are shown inside each part of the individual bar plots. *IGFBP7* expression as a continuous variable by pCR in **C** the ganitumab/metformin plus chemotherapy arm and in **D** the chemotherapy-alone control arm. Violin plots illustrating the distribution of *IGFBP7* expression by pCR status with overlaying box plots. In the box plots, the boundary of the box closest to zero indicates the 25th percentile, a black line within the box marks the median, and the boundary of the box farthest from zero indicates the 75th percentile. Points above the whiskers (Q3 + 1.5*interquartile range (IQR)) and below the (Q1−1.5*interquartile range (IQR)) indicate outliers. Fig. panels **A**–**D** are descriptive, and no statistical analyses were performed. The main statistical analyses for the associations between pCR rates and *IGFBP7* expression by treatment arm were performed using logistic regression and are presented in Tables 2–4.

In the ganitumab/metformin plus chemotherapy arm, 22.6% of patients achieved a pCR compared to 16.8% in the chemotherapy-alone control arm, as previously reported^13^, and the difference was not considered clinically significant. We observed that the efficacy of ganitumab/metformin plus chemotherapy treatment in achieving pCR was modified by *IGFBP7* expression, with interactions in the crude and multivariable models (all *P* ≤ 0.017; Table 2). As shown in Fig. 1, our analysis revealed that the expression level of *IGFBP7* strongly predicted the probability of achieving pCR in patients in the ganitumab/metformin plus chemotherapy arm (adjusted OR 0.38 [95% CI 0.17–0.80]) but not in the chemotherapy-alone control arm (adjusted OR 1.23 [95% CI 0.63–2.45], *P*_interaction_ = 0.016; Figs. 1, 2D, E, and Table 2). The probability of achieving pCR declined dramatically with increasing expression of IGFBP7, with pCR rates of 46.9%, 17.2%, 11.5%, and 5.6% across quartiles of increasing IGFBP-7 expression. Quartiles (Q)2-4 of *IGFBP7* expression compared to Q1 conferred lower likelihood of achieving pCR in the ganitumab/metformin plus chemotherapy arm (adjusted OR 0.09 [95% CI 0.00–0.58]), but not in the chemotherapy-alone control arm (adjusted OR 1.38 [95% CI 0.41–4.73], *P*_interaction_ = 0.031; Fig. 2B, C). In the chemotherapy-alone control arm, pCR rates did not vary between quartiles of *IGFBP7* expression. The crude and adjusted ORs for *IGFBP7* expression modeled as both a continuous variable and quartiles and other clinicopathological factors are provided in Table 2.

**Table 2:** Odds ratio of achieving a pCR in relation *IGFBP7* expression in chemotherapy-alone control arm and ganitumub + metformin arm

	IGFBP7 continuous		IGFBP7 quartiles		
	Chemotherapy-alone control	Ganitumab + metformin	Chemotherapy-alone control	Ganitumab+ metformin	
	OR (95% CI)	OR (95% CI)	OR (95% CI)	OR (95% CI)	
**Crude**					**Interactions**
IGFBP7 Continuous	1.05 (0.58, 1.95)	0.32 (0.15, 0.64)			LR *P* = 0.011
IGFBP7 Quartiles					LR *P* = 0.011
Q1			Ref.	Ref	
Q2			1.54 (0.50, 4.80)	0.24 (0.07, 0.74)	
Q3			1.23 (0.39, 3.91)	0.15 (0.03, 0.53)	
Q4			1.19 (0.38, 3.80)	0.07 (0.00, 0.39)	
**Multivariable**					**Interactions**
IGFBP7 Continuous	1.23 (0.63, 2.45)	0.38 (0.17, 0.80)			LR *P* = 0.017
IGFBP7 Quartiles					LR *P* = 0.015
Q1			Ref.	Ref.	
Q2			1.62 (0.51, 5.28)	0.27 (0.07, 0.92)	
Q3			1.86 (0.53, 6.65)	0.17 (0.03, 0.69)	
Q4			1.38 (0.41, 4.73)	0.09 (0.00, 0.58)	
ER/PgR+	1.44 (0.57, 3.64)	0.64 (0.16, 2.38)	1.38 (0.54, 3.56)	0.56 (0.14, 2.15)	
MP2	1.85 (0.65, 5.70)	1.40 (0.20, 10.0)	1.81 (0.63, 5.57)	1.27 (0.19, 9.02)	
Immune+	1.00 (0.35, 2.82)	1.88 (0.58, 6.33)	1.06 (0.37, 3.00)	1.77 (0.52, 6.19)	
DRD+	2.35 (0.67, 7.84)	1.84 (0.43, 8.32)	2.43 (0.76, 8.24)	1.91 (0.43, 8.91)	

Control: Paclitaxel followed by anthracyclines.

*OR* odds ratio, *CI* confidence Interval, *LR* likelihood ratio.

We then stratified patients by breast cancer subtype, high-risk estrogen receptor (ER), and/or progesterone receptor (PgR)-positive/human epidermal growth factor receptor 2 (HER2)-negative versus triple-negative breast cancer (TNBC). The ability of *IGFBP7* expression to identify breast cancers more likely to respond to ganitumab/metformin plus chemotherapy than to chemotherapy alone was most apparent in TNBC, where 66.7% of *IGFBP7* Q1 tumors and no *IGFBP7* Q4 tumors achieved pCR (Table 3). In an exploratory analysis, stratified by *IGFBP7* expression quartiles, ganitumab/metformin plus chemotherapy treatment conferred a higher likelihood of achieving pCR in tumors with low *IGFBP7* expression (Q1-2) (adjusted OR 2.64 [95% CI 1.16–5.49]), but not in tumors with high *IGFBP7* expression (Q3-4) (adjusted OR 0.44 [95% CI 0.11–1.40]). The improved efficacy of ganitumab/metformin plus chemotherapy treatment compared to standard chemotherapy in achieving pCR was confined to the patients in the lowest quartile of *IGFBP7* expression (Fig. 1 and Table 4).

**Table 3:** pCR in relation *IGFBP7* expression in the chemotherapy-alone control arm and chemotherapy/ganitumab/metformin arm stratified by receptor subtype

	ERR/PgR + HER2‒		TNBC	
	Chemotherapy-alone	Ganitumab + metformin	Chemotherapy-alone	Ganitumab + metformin
	**pCR/Total (%)**	**pCR/Total (%)**	**pCR/Total (%)**	**pCR/Total (%)**
All patients	14/92 (15.2%)	8/57 (14.0%)	15/83 (18.1%)	16/46 (34.8%)
*IGFBP7* quartiles				
Q1	5/21 (23.8%)	3/14 (21.4%)	2/29 (6.9%)	12/18 (66.7%)
Q2	3/21 (14.3%)	2/16 (12.5%)	5/19 (26.1%)	3/13 (23.1%)
Q3	2/31 (6.4%)	2/15 (13.3%)	5/11 (45.5%)	1/10 (10%)
Q4	4/19 (21.0%)	1/12 (8.3%)	3/24 (12.5%)	0/5 (0%)
	**OR** **(95% CI)**	**OR** **(95% CI)**	**OR** **(95% CI)**	**OR** **(95% CI)**
*IGFBP7* continuous	0.69 (0.24, 1.94)	0.57 (0.16, 1.85)	1.35 (0.64, 3.05)	0.28 (0.09, 0.67)

In TNBC, the association between high IGFBP7 gene expression and low pCR rates was especially pronounced in the chemotherapy/ganitumab/metformin arm.

*OR* odds ratio, *CI* confidence interval, *HR* hormone receptor, *HER2* human epidermal growth factor receptor 2, *TNBC* triple-negative breast cancer.

**Table 4:** Odds ratio of achieving a pCR in the chemotherapy/ganitumab/metformin arm compared to the chemotherapy-alone control arm, stratified by quartile and median of *IGFBP7* gene expression, I-SPY2

		*IGFBP7* expression			
	All patients	Q1	Q2	Q3	Q4
**Variable**	**OR** **(95% CI**)	**OR** **(95% CI**)	**OR** **(95% CI**)	**OR** **95% CI**	**OR** **95% CI**
**Crude**					
Trial arm					
Chemotherapy-alone	Ref.	Ref.	Ref.	Ref.	Ref.
Ganitumab + metformin	1.45 (0.79, 2.65)	5.42 (1.94, 16.5)	0.83 (0.23, 2.82)	0.65 (0.13, 2.61)	0.30 (0.02, 1.90)
**Multivariable**					
Trial arm					
Chemotherapy-alone	Ref.	Ref.	Ref.	Ref.	Ref.
Ganitumab + metformin	1.50 (0.79, 2.81)	6.17 (2.01, 21.3)	0.84 (0.22, 3.05)	0.35 (0.06, 1.69)	0.25 (0.01, 2.55)
ER/PgR+	1.07 (0.52, 2.18)	0.74 (0.22, 2.42)	1.00 (0.23, 4.11)	0.00	8.05 (1.25, 75.3)
MP2	2.12 (0.92, 5.14)	0.85 (0.20, 4.13)	4.00 (0.57, 38.1)	0.00	1.74 (0.19, 20.8)
Immune+	1.22 (0.58, 2.57)	0.77 (0.16, 3.30)	0.89 (0.19, 4.08)	12.7 (1.46, 285)	0.56 (0.06, 4.74)
DRD+	2.09 (0.90, 4.99)	4.59 (1.02, 25.0)	1.44 (0.26, 8.58)	0.04 (0.00, 0.71)	23.0 (1.43, 900)
		Low (Q1–2)		High (Q3–4)	
		OR (95% CI)		OR (95% CI)	
**Crude**					
Trial arm					
Chemotherapy-alone	Ref.	Ref.	Ref.		
Ganitumab + Metformin	1.45 (0.79, 2.65)	2.50 (1.16, 5.49)	0.49 (0.13, 1.49)		
**Multivariable**					
Trial arm					
Chemotherapy-alone	Ref.	Ref.	Ref.		
Ganitumab + Metformin	1.50 (0.79, 2.81)	2.64 (1.17, 6.10)	0.44 (0.11, 1.40)		
ER/PgR+	1.07 (0.52, 2.18)	0.83 (0.33, 2.01)	1.61 (0.41, 6.60)		
MP2	2.12 (0.92, 5.14)	1.72 (0.55, 6.03)	3.23 (0.74, 16.6)		
Immune+	1.22 (0.58, 2.57)	0.92 (0.33, 2.53)	2.57 (0.72, 9.69)		
DRD+	2.09 (0.90, 4.99)	2.50 (0.85, 7.81)	1.10 (0.24, 5.01)		

*OR* odds ratio, *CI* confidence interval.

### *IGFBP7* gene expression in relation to survival

In the SCAN-B study, the median follow-up for the 4158 patients still at risk was 5.45 (interquartile range 5.07–8.15) years and descriptive statistics are presented in Supplementary Table 3. The included and excluded patients in the SCAN-B cohort are presented in Supplementary Fig. 1. In the univariable survival analyses of *IGFBP7* expression in biopsied breast cancer tissue in the SCAN-B cohort, *IGFBP7* expression, Q4 compared to Q1, was not associated with increased risk of recurrence (HR 0.96 [95% CI 0.76–1.22]) or distant metastasis (HR 1.00 [95% CI 0.77–1.32], Supplementary Table 4). However, after adjustment for age, clinicopathological factors, and treatments in the multivariable models, high *IGFBP7* expression, Q4 compared to Q1, was associated with a significantly increased risk of recurrence (HR 1.37 [95% CI 1.04–1.82]) and distant metastasis (HR 1.60 [95% CI 1.15–2.22], Supplementary Table 4). When modeled as a continuous variable, *IGFBP7* expression showed similar associations with clinical outcome (Supplementary Table 5). Most importantly, when modeled as a continuous variable, *IGFBP7* expression in the adjusted model also conferred a significantly increased risk of recurrence (HR 1.29 [95% CI 1.09–1.53]) and distant metastasis (HR 1.41 [95% CI 1.16–1.73], Supplementary Table 5).

### *IGFBP7* gene expression in relation to molecular features, immune signatures, and tumor microenvironment composition

**Fig. 3:**
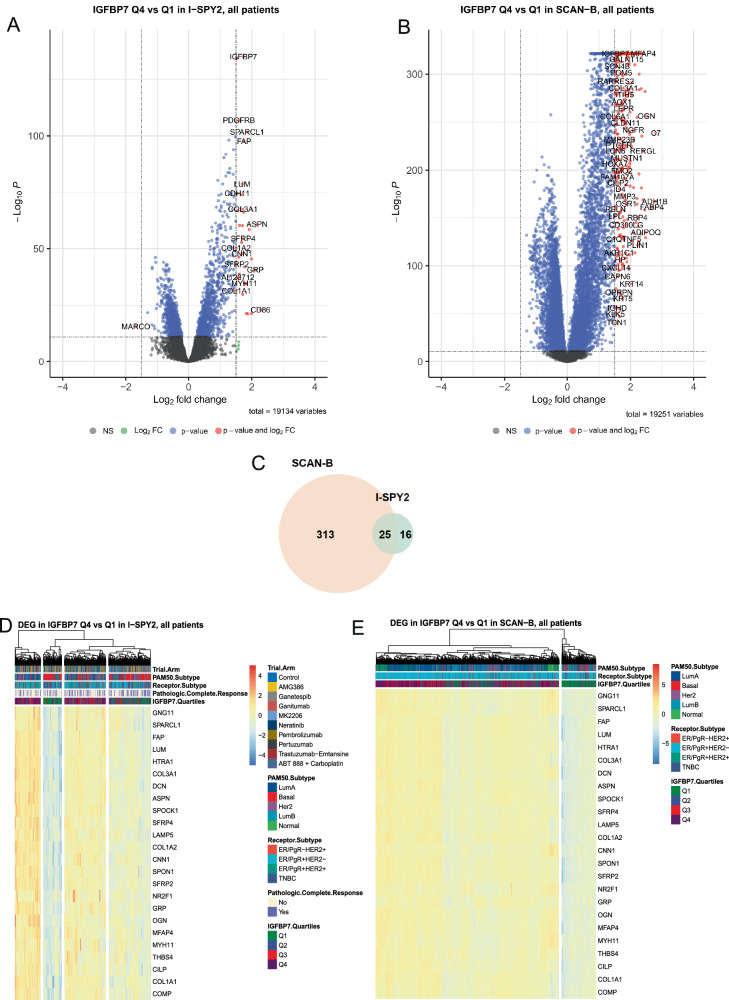
Molecular analyses of *IGFBP7* expression in I-SPY2 and SCAN-B. Volcano plots showing significantly up- and downregulated genes in *IGFBP7* Q4 compared to in *IGFBP7* Q1 tumors from the differential gene expression (DGE) analysis in ‘Limma-Voom’ ranked by false discovery rate (FDR)-adjusted *P*-values (*x*-axis) and fold change (log2FC; *y*-axis) FC criteria in **A** I-SPY2 and **B** SCAN-B. Differentially expressed genes (DEG) are defined as FDRkkkk of ≤ 0.05 and fold change (log2FC) log2FC ≥ 1.5 for up-regulated genes and log2FC ≤ −1.5 and indicated by red. Blue indicates genes fulfilling the FDR criteria but not the log2FC criteria. Green indicates genes fulfilling the log2FC criteria but not the FDR criteria. Gray indicates genes not fulfilling either criterion. **C** Venn diagram of genes that were differentially expressed in both I-SPY2 and SCAN-B. Heatmap of the 25 overlapping DEGs from the DGE analysis in ‘Limma-Voom’ ranked by fold change (log2FC) in *IGFBP7* Q4 compared to in *IGFBP7* Q1 tumors in (**D**) I-SPY2 and (**E**) SCAN-B. Tumor samples are in columns and biomarkers are in rows. Red indicates higher expression and blue lower expression. Annotation tracks reflect pCR (purple), receptor subtype, PAM50 subtype, and trial arm (light red: ganitumab/metformin plus chemotherapy). See Supplementary Tables 6–9 for more information.

Having demonstrated that low *IGFBP7* expression is predictive of a benefit of adding ganitumab to chemotherapy in neoadjuvant breast cancer treatment and that high *IGFBP7* expression predicts poor outcome, we next sought to determine if *IGFBP7* expression was related to other breast cancer characteristics. In both ISPY-2 and SCAN-B, *IGFBP7* expression was positively correlated with *IGFBP3-6* and *IGF1* and *IGF2* expression (all *r*_s_ ≥ 0.14; Supplementary Fig. 2A, B). The correlations between *IGFBP7* expression and the eight gene modules were similar in ISPY-2 and SCAN-B. *IGFBP7* expression was positively correlated with stroma, lipid, and early response to growth signaling modules (all *r*_s_ ≥ 0.21) and negatively correlated with mitotic checkpoint and progression modules (all *r*_s_ ≤ −0.10; Supplementary Fig. 2C, D). The correlations suggest an association with aggressive tumor microenvironment (TME) and increased growth factor signaling activation. Likewise, *IGFBP7* expression was highest in normal-like, indicating stromal activation, followed by luminal A subtype in both cohorts (both *P* < 0.001; Supplementary Fig. 2E, F). *IGFBP7* expression did not vary across ER/PgR-positive and HER2-positive subtypes but was somewhat lower in TNBC (both *P* < 0.001; Supplementary Fig. 2G, H). *IGFBP7* expression was only correlated with the mast cell signature in both I-SPY2 and SCAN-B and clustered far from the main immune components (Supplementary Fig. 2C, D and Fig. 3A–C). This finding suggests that IGFBP7 is neutral with regards to immune activation in breast tumors.

Differential gene expression (DGE) analyses were performed comparing *IGFBP7* Q4 versus Q1 tumors in I-SPY2 and SCAN-B with a total of 25 genes found to be differentially expressed in both cohorts (Fig. 3A–C). Notably, higher expression of several genes coding for proteins involved in endothelial cell regulation and extracellular matrix remodeling, *e.g. COMP, FAP, COL1A3, OGN, LUM, COL1A1, SPOCK1, COL1A2, DCN*, and *SPON1* were seen in *IGFBP7* Q4 tumors compared to Q1 tumors, supporting a potential association with an active TME (Fig. 3A-B and Supplementary Tables 6–8). Furthermore, *IGFBP7* Q1 tumors were clearly defined by low or absent expression of these genes, implying that the stromal component of the tumor is crucial for *IGFBP7* expression (Supplementary Fig. 4A, B). Significantly enriched signature Hallmarks in *IGFBP7* Q4 tumors in both I-SPY2 and SCAN-B included EMT, angiogenesis. coagulation, and transforming growth factor beta (TGF-β) signaling (Supplementary Fig. 4C, D and Tables 9–11). The leading-edge subsets, the genes driving the enrichment, were the most similar for EMT, angiogenesis, TGF-β, and IL2/STAT5 signaling with Jaccard indices over 55% (Supplementary Fig. 4C, D and Tables 9, 10). Network analysis showed that these gene subsets belonged mostly to EMT driving the enrichment scores of the other hallmarks (Supplementary Table 11). This finding suggests a key role of *IGFBP7* in EMT.

**Fig. 4:**
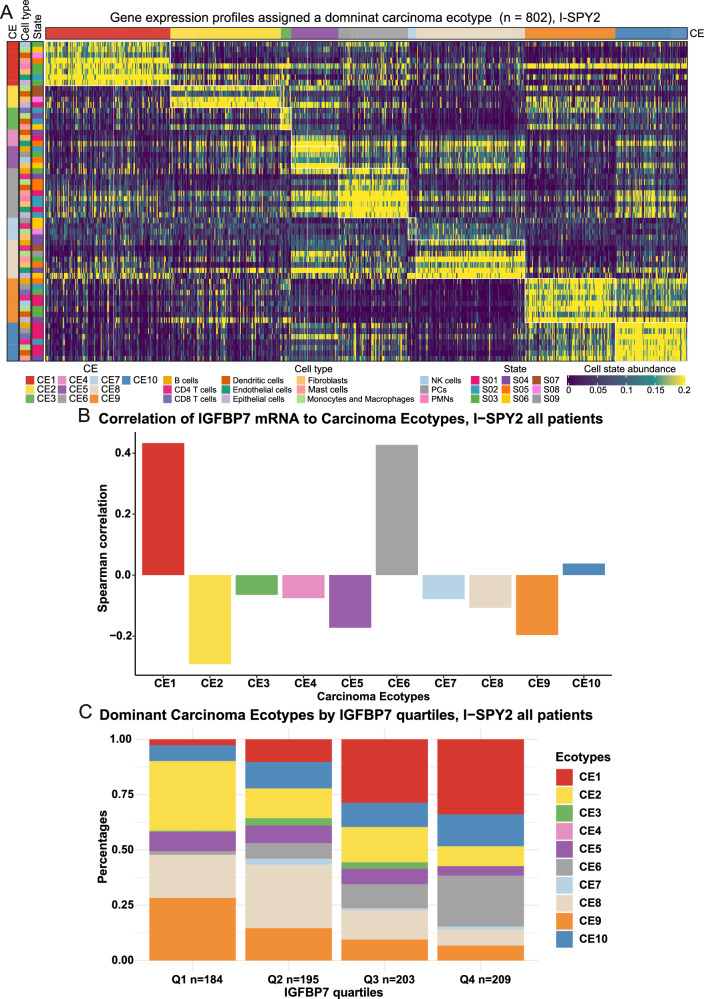
Tumor microenvironment composition in relation to *IGFBP7* gene expression. Heatmap **A** of carcinoma ecotypes (CE), cell types, and cell states in patients (across all arms) in I-SPY2 whose tumors were assigned a dominant ecotype. Cell-state abundance profiles across I-SPY2, organized into ten CEs. Only cell states and tumor samples assigned to CEs are shown. Tumor samples are ordered by the most abundant CE class per specimen. Bar plots of Pearson correlations coefficients **B** of *IGFBP7* gene expression as a continuous variable and relative abundance of each CE calculated across the entire biomarker study in I-SPY2. The 10 CEs are presented on the *x*-axis, and the Pearson correlation values are presented on the *y*-axis. The dominant CE (**C**) in breast cancers by *IGFBP7* quartiles. The *y*-axis indicates the percentage of dominant CE in tumors, and *IGFBP7* quartiles are indicated on the *x*-axis. The Chi-square test was used for statistical analysis in panel **C**.

Tumor tissue composition in relation to *IGFBP7* expression was estimated by ECOTYPER^30^. High *IGFBP7* gene expression was associated with dominance of Carcinoma Ecotype (CE)6, CE1, followed by CE10 (*P* < 0.001; Fig. 4A, B). Tumors with high *IGFBP7* gene expression had a microenvironment enriched for stromal cells while deficient in immune cells, characterized by transforming growth factor beta signaling, indicating enrichment of cancer-associated fibroblasts and aging tissue features (Fig. 4A). Conversely, tumors with low *IGFBP7* gene expression were dominated by CE2 and CE9, indicating basal-like features and a pro-inflammatory response (Fig. 4C).

## Discussion

We report, using data from the I-SPY trial, that breast cancer patients with tumors showing low *IGFBP7* gene expression were more than twice as likely to achieve a pCR with neoadjuvant treatment using the combination of ganitumab/metformin, and chemotherapy compared to neoadjuvant chemotherapy alone. In contrast, *IGFBP7* expression was unrelated to the probability of achieving a pCR with chemotherapy alone. To our knowledge, this is the first study that identifies a specific biomarker related to efficacy of IGF-1R targeting treatment. By the use of *IGFBP7* as a predictor of ganitumab efficacy, we identified a substantial proportion of HER2 negative breast cancers (approximately 25%) including both poor prognosis ER/PgR-positive HER2-negative cancers and TNBC where the addition of ganitumab/metformin plus chemotherapy substantially improves pCR rates.

Our findings motivate laboratory research to uncover the biology underlying our observations. *IGFBP7* expression might directly influence ganitumab efficacy by competing with the antibody for binding to IGF-1R, but it is also possible that *IGFBP7* expression serves as a marker of a more aggressive neoplastic phenotype^26,27^. These possibilities are not mutually exclusive. Our observation that that high *IGFBP7* expression was associated with worse clinical outcome in SCAN-B, a large contemporary population-based breast cancer cohort unexposed to ganitumab, supports the concept that high *IGFBP7* expression reveals more than increased resistance to ganitumab.

Our finding that low *IGFBP7* expression defines a subset of patients where the addition of ganitumab to chemotherapy approximately doubles the pCR rate, shows the utility of the I-SPY2 trial design. Furthermore, our results demonstrate that detailed examination of the publicly available I-SPY2-990 Data Resource, either hypothesis-driven (as in this case), or agnostically, can provide opportunities to test hypotheses that were not proposed before or during the execution of the trial. The initial assessment of IGF signaling axis in the I-SPY2 ganitumab arm^13^ was negative but failed to include IGFBP7, a non-canonical IGFBP. Had *IGFBP7* been included in the initial assessment of predictive biomarkers in I-SPY2, it would have identified the distinct subgroup where ganitumab showed benefit. Our findings add to the list of important studies^18,20–23^ where the I-SPY2 trial detected activity of novel agents^17,18,22,23^, even if the benefit is shown to be confined to a biomarker-defined subset of patients.

The molecular features of tumors with high *IGFBP7* expression suggest an aggressive TME that facilitates metastasis, potentially through EMT. The ECOTYPER data corroborate the notion that *IGFBP7* expression may be used to classify distinct subtypes of the breast cancer microenvironment. This is consistent with previous studies showing that both systemic and tumor-specific IGFBP7 protein levels were poor prognostic markers in breast cancer^28,29^, and are also in line with our results from SCAN-B.

Despite early studies^24,26–29^, there are many gaps in knowledge concerning the manner by which IGFBP7 modulates signaling by the IGF-1R family, and potentially the efficacy of anti-IGF-IR antibodies. Our findings provide a strong rationale for further experimental work to elucidate how IGFBP7 diminishes the effect of ganitumab and biological influence of IGFBP7 on the TME. The limited evidence available indicates a potential role of IGFBP7 in PI3K/Akt signaling, where it promotes increased activation^26,27^. Obesity is considered to promote PI3K/Akt signaling through mechanisms involving increased levels of insulin and leptin, and possible relationships between obesity, *IGFBP7* expression, and ganitumab sensitivity deserve further study^31,32^.

IGFBP7 merits further study as a treatment predictive biomarker in other cancer types such as colon, ovarian and prostate cancer, as well as sarcomas, where IGF-1R targeting agents have been investigated^9,33^. We emphasize that the lack of data concerning HER2-positive breast cancers does not imply lack of activity in these patients; they simply were not eligible for the ganitumab arm of the I-SPY2 study because of non-availability of clinical safety data regarding co-administration of ganitumab and HER2 targeting agents. In fact, prior literature^34^ has suggested benefit of co-targeting IGF-IR and HER2 signaling.

There are few treatment options beyond chemotherapy in TNBC. Recently, immune checkpoint inhibitors were introduced in clinical practice for treatment of high-risk TNBC^17,18,35,36^. This has led to great interest in the immune microenvironment of breast cancer. Our data indicate that *IGFBP7* expression is either neutral to immune activation or is positively correlated with immune depletion in the tumor depending on whether immune gene signatures or ECOTYPER was used to profile the immune components of the tumors. Further studies are needed to elucidate the potential role of IGFBP7 in the context of breast cancer immunotherapy.

Previous in vitro work demonstrated that IGF-1R stimulates TNBC cell proliferation and survival through the Ras/ERK or PI3K/Akt pathways, showing that IGF-1R signaling is important in this context^37^. A substantial proportion of TNBC could have improved outcomes with the addition of ganitumab/metformin to chemotherapy, provided that low IGFBP7 is validated as a predictive biomarker. Ganitumab/metformin also has a fairly benign side effect profile^13^ and could address an unmet need for TNBC. The main side effect of ganitumab is temporary treatment-induced hyperglycemia, which can be managed with metformin. However, it has been demonstrated that this glucose–insulin feedback to treatment reactivates PI3K/Akt signaling even in the presence of a PI3K inhibitor^38–40^. A ketogenic diet and sodium-glucose cotransporter-2 (SGLT2) inhibitors decrease the glucose–insulin feedback response^38–40^, while metformin does not. Circulating IGFBP7 levels can identify patients who derive the greatest benefit from SGLT2 inhibitors in treating cardiovascular disease^41^. Whether *IGFBP7* expression can identify patients where SGLT2 inhibitors decrease the glucose–insulin feedback loop by impacting *IGFBP7* expression merits further study.

The strengths of this study include the use of contemporary data from both the I-SPY2 trial and the SCAN-B cohort. SCAN-B allows for the evaluation of biomarkers in a contemporary real-world setting due to the large-scale RNAseq analysis of consecutively enrolled breast cancer patients^42–44^. Another strength is that I-SPY2 evaluates ganitumab in a randomized controlled setting, providing an ideal platform for investigation of treatment-specific biomarkers for ganitumab: the impact of *IGFBP7* expression on the response to ganitumab with chemotherapy is more convincing in the light of the contrasting irrelevance of *IGFBP7* expression to response to chemotherapy alone. Finally, *IGFBP7* expression showed remarkably stable associations with clinicopathological factors and molecular features in the completely independent SCAN-B and ISPY-2, studies, giving evidence of reproducibility of these associations.

The current study, while hypothesis-driven, is a retrospective analysis of existing datasets, and thus, the results require further validation in prospective studies. Another limitation is the lack of data on long-term outcomes in I-SPY2, but it has been previously shown that pCR has a strong association with distant metastasis-free survival^45^. Due to the combination of ganitumab/metformin plus chemotherapy in the treatment arm, it is not possible to determine with certainty whether *IGFBP7* expression identifies tumors sensitive to ganitumab alone, metformin alone, or the combination. However, the MA.32 randomized clinical trial did not show the benefit of adjuvant metformin compared to standard therapy in early-stage breast cancer^46^, suggesting that the observed effect in I-SPY2 was driven by ganitumab. It must also be acknowledged that certain biomarker-defined subgroups are relatively small, which leads to statistical imprecision. Unfortunately, when analyzing biomarkers in a clinical trial, small subgroups can occur since exploratory biomarkers are not considered when determining the pre-planned trial size. The main drawback of the study is the relatively short follow-up in SCAN-B, especially for ER-positive disease. This trade-off occurs when using more contemporary cohorts as the follow-up has not yet matured. However, at the same time, long follow-up means that treatment regimens have changed. The results obtained from older cohorts with longer follow-up may, therefore, not be generalizable to contemporary breast cancer patients.

In conclusion, low *IGFBP7* gene expression identifies a subset of breast cancer patients for whom the addition of ganitumab/metformin to neoadjuvant chemotherapy results in a significantly improved pCR rate compared to neoadjuvant chemotherapy alone. This justifies laboratory studies to address gaps in knowledge concerning the roles of IGFBP7 in neoplasia, and the relevance of this protein to IGF-1R-targeting agents. Such research, together with the results presented here, may lead to a review of decisions to halt the development of IGF-1R-targeting drugs, which were based on disappointing results of prior trials that did not use predictive biomarkers.

## Methods

### I-SPY2

The I-SPY2 trial (NCT01042379) is an open-label, multicenter, adaptively randomized phase 2 trial of neoadjuvant therapy, evaluating multiple investigational arms in parallel. Neoadjuvant paclitaxel followed by doxorubicin/cyclophosphamide with trastuzumab in HER2+ disease serves as the common chemotherapy-alone control arm^22,23^. Investigational agents are combined with this regimen^22,23^. The primary endpoint is pCR, defined as ypT0/Tis, ypN0^22,23^. Eligibility for I-SPY2 includes age 18 years or older, stage II or III breast cancer, and primary tumor size >2.5 cm by clinical examination or >2.0 cm by imaging^22,23^. Patients with ER/PgR-positive cancers are eligible only if they have a poor prognosis estimated by the MammaPrint (MP) 70-gene-based prognostic signature^20^. Subsequent adjuvant endocrine treatment was at the discretion of the treating physician. Ganitumab was tested in combination with metformin since ganitumab can induce hyperglycemia^13^. In the I-SPY2 study of the IGF-IR blocking antibody ganitumab, patients with HER2+ tumors were not included since there is no safety data concerning the combination of ganitumab and trastuzumab^13^. The ganitumab/metformin plus chemotherapy arm only included TNBC patients and patients who were ER/PgR-positive and HER2-negative in their tumors as long as their MammaPrint gene signature indicated poor prognosis. Gene expression profiling of core needle biopsies taken from the primary tumor before treatment was performed using Agilent 44K expression arrays^20^. Transcriptomic and clinical data of patients enrolled in the I-SPY2 trial were obtained from the Gene Expression Omnibus (GEO) database (GSE194040). For the analysis reported here, we studied patients from the ganitumab/metformin plus chemotherapy arm and the chemotherapy-alone control arm, excluding patients with HER2+ disease (Fig. 1).

### SCAN-B

The Swedish Cancerome Analysis Network—Breast (SCAN-B; NCT02306096) is an ongoing population-based cohort. SCAN-B prospectively includes breast cancer patients diagnosed and treated at nine Swedish hospitals^42,43^. All newly diagnosed breast cancer patients are invited to participate^43^. Tumor specimens or core needle biopsies in case of neoadjuvant treatment from the patients’ tumors are obtained in conjunction with routine clinical sampling^42,43^. The samples are subject to gene expression profiling using RNA-seq according to custom SCAN-B workflow^42–44^. Gene expression levels were expressed in fragments per kilobase of exon per million mapped reads (FPKM)^44^. Clinical data was collected from the Swedish National Quality Registry for Breast Cancer^42–44^. Curated RNA-seq and clinical data were accessed from Staaf et al. ^44^. A subset of 5326 patients with available follow-up for distant metastasis, no bilateral cancer, and gene expression profiles (GEXs) from primary invasive breast cancer was analyzed (Supplementary Fig. 1). If multiple GEXs for a single patient were available, the GEX with the highest RNA concentration was chosen^44^, leaving one GEX per patient for analysis.

### Data analysis

In both I-SPY2 and SCAN-B, eight gene expression modules representing different biological functions and different immune gene signatures in breast cancer were calculated as previously described^17,47^. The correlation coefficients between *IGFBP7* gene expression, gene expression of 15 other proteins involved in the IGF/Insulin pathway, immune gene signatures, and the eight modules were calculated using Pearson’s correlation. Differences in *IGFBP7* gene expression depending on the PAM50 subtype and receptor subtype were compared using the Kruskal–Wallis test. The Chi-square test was used to test associations between categorical variables.

In the regression analyses, *IGFBP7* gene expression was modeled both as a continuous variable and categorized as quartiles; quartile 1 (Q1), quartile 2 (Q2), quartile 3 (Q3), and quartile 4 (Q4), to allow for non-linear effects. The lowest expression of *IGFBP7* (Q1) was used as a reference. Quartiles were created separately for I-SPY2 and SCAN-B based on the entire datasets.

Crude and adjusted odds ratios (OR) with 95% confidence intervals (CI) for pCR in I-SPY2 were estimated using logistic regression. The multivariable models were adjusted a priori for potential confounders either previously known factors associated with likelihood of achieving pCR and/or variables that somewhat differed by *IGFBP7* quartiles: ERR/PgR+, HER2+, trial arm with chemotherapy-alone control arm as reference, PAM50 subtype with luminal A as reference, MP ultra-high risk (MP2), ISPY-2 immune (Immune+), and DNA repair deficiency (DRD+) signatures^20^. To investigate if there was any interaction between *IGFBP7* gene expression and efficacy of ganitumab/metformin plus chemotherapy treatment in achieving pCR, interaction analyses were performed in the ganitumab/metformin plus chemotherapy arm and the HER2 negative subset of the chemotherapy-alone control arm. The interaction was tested in both crude and adjusted models by including an interaction term and comparing the models with or without the interaction term using the Likelihood ratio (LR) test. The *P*-values for the interaction term and LR test are reported. Due to sparse data, the PAM50 subtype could not be included in the interaction analyses.

The endpoints used for survival analyses in SCAN-B were recurrence-free interval (RFI) and distant metastasis-free interval (DMFI)^44^. Cox proportional hazard regression was used to estimate crude and adjusted hazard ratios (HRs) with 95% CI. The multivariable models were adjusted for a priori selected standard clinicopathological factors: age (binned in 5-year intervals), tumor characteristics; lymph node status (pN1/2/3), tumor size (pT2/3/4), Grade (III), ER+, PgR+, HER2+, PAM50 subtype with luminal A as reference, PAM50 ROR category i.e., High versus Low/Intermediate; and (neo)adjuvant treatments; endocrine treatment, chemotherapy, and trastuzumab.

Differential gene expression (DGE) analysis was performed using the ‘Limma-Voom’ package^48^. The criteria used to define differentially expressed genes (DEGs) between the Q4 and Q1 of *IGFBP7* expression was false discovery rate (FDR) of ≤ 0.05 and log2-fold change (log2FC) ≥ 1.5 for up-regulated genes and log2FC ≤ −1.5 for down-regulated genes. The ‘clusterprofiler’^49^ package was used to perform gene set enrichment analysis (GSEA). Gene sets were grouped according to Hallmark Signatures^50^. The results from the separate analyses were compared to find similar gene expression patterns between I-SPY2 and SCAN-B datasets. A leading-edge analysis was performed separately for each dataset to find the genes that were driving the hallmark enrichment. The leading-edge results for the hallmarks found to be activated or suppressed in both I-SPY2 and SCAN-B were compared for similarity using the Jaccard index. The results were visualized using ‘EnhancedVolcanoplot’, ‘pHeatmap’, ‘Circlize’, ‘VennDiagram’, and ‘clusterprofiler’^49^ packages. In silico profiling of different carcinoma ecosystems, including estimates of relative abundance, were derived from gene expression profiles of tumors from I-SPY2 using a deconvolution-based method, ECOTYPER, with standard parameters^30^. ECOTYPER applies a machine learning framework for large-scale identification of cell states and cellular ecosystems from bulk gene expression data^30^.

All data analyses were conducted in R version 4.2.2. *P*-values < 0.05 were considered statistically significant. All *P*-values were two-sided. This study followed the Reporting Recommendations for Tumor Marker Prognostic Studies (REMARK) criteria^51^.

### Ethics

Ethical approvals for I-SPY2 and SCAN-B were obtained in relation to the primary projects and publications^20,22,23,42–44^. The I-SPY2 trial was approved by each respective institutional review board of the participating, and SCAN-B was approved by the Lund University Ethics Committee. No other separate approval was obtained for this specific study since it is based on publicly available data. All participants signed written informed consent. The study was conducted in accordance with the ethical principles of the Declaration of Helsinki.

## Supplementary information

Supplementary Figures and Tables

Supplementary Tables 6-13

## Data Availability

Clinical and RNA-seq data from SCAN-B are accessible from Staaf et al. ^44^, available at Mendeley Data https://data.mendeley.com/datasets/yzxtxn4nmd/3. The clinical and transcriptomic data from I-SPY2 are available from the GEO database (http://www.ncbi.nlm.nih.gov/geo/) under accession identification number GSE194040.

## Code availability

All analyses were performed using the R statistical environment, an open-source software. This paper does not report the original code. Any code or additional information required to reanalyze the data reported in this paper is available upon request.

## References

[1] Pollak, M. The insulin and insulin-like growth factor receptor family in neoplasia: an update. *Nat. Rev. Cancer* **12**, 159–169 (2012). 22337149 10.1038/nrc3215

[2] Sachdev, D. & Yee, D. Disrupting insulin-like growth factor signaling as a potential cancer therapy. *Mol. Cancer Ther.* **6**, 1–12 (2007). 17237261 10.1158/1535-7163.MCT-06-0080

[3] Huff, K. K. et al. Secretion of an insulin-like growth factor-I-related protein by human breast cancer cells. *Cancer Res.* **46**, 4613–4619 (1986). 3731113

[4] Luey, B. C. & May, F. E. Insulin-like growth factors are essential to prevent anoikis in oestrogen-responsive breast cancer cells: importance of the type I IGF receptor and PI3-kinase/Akt pathway. *Mol. cancer* **15**, 1–15 (2016). 26801096 10.1186/s12943-015-0482-2 PMC4722749

[5] King, H., Aleksic, T., Haluska, P. & Macaulay, V. M. Can we unlock the potential of IGF-1R inhibition in cancer therapy? *Cancer Treat. Rev.* **40**, 1096–1105 (2014). 25123819 10.1016/j.ctrv.2014.07.004 PMC4196677

[6] Vashisth, H. Theoretical and computational studies of peptides and receptors of the insulin family. *Membranes* **5**, 48–83 (2015). 25680077 10.3390/membranes5010048 PMC4384091

[7] Xu, Y. et al. How IGF-II binds to the human type 1 insulin-like growth factor receptor. *Structure* **28**, 786–798. e786 (2020). 32459985 10.1016/j.str.2020.05.002 PMC7343240

[8] Li, J., Choi, E., Yu, H. & Bai, X.-c. Structural basis of the activation of type 1 insulin-like growth factor receptor. *Nat. Commun.* **10**, 4567 (2019). 31594955 10.1038/s41467-019-12564-0 PMC6783537

[9] Ekyalongo, R. C. & Yee, D. Revisiting the IGF-1R as a breast cancer target. *NPJ Precis. Oncol.* **1**, 14 (2017). 29152592 10.1038/s41698-017-0017-y PMC5687252

[10] Paez, J. G. et al. EGFR mutations in lung cancer: correlation with clinical response to gefitinib therapy. *Science* **304**, 1497–1500 (2004). 15118125 10.1126/science.1099314

[11] Sequist, L. V. et al. First-line gefitinib in patients with advanced non-small-cell lung cancer harboring somatic EGFR mutations. *J. Clin. Oncol.* **26**, 2442–2449 (2008). 18458038 10.1200/JCO.2007.14.8494

[12] Harbeck, N. et al. Breast cancer. *Nat. Rev. Dis. Prim.* **5**, 66 (2019). 31548545 10.1038/s41572-019-0111-2

[13] Yee, D. et al. Ganitumab and metformin plus standard neoadjuvant therapy in stage 2/3 breast cancer. *NPJ Breast Cancer* **7**, 131 (2021). 34611148 10.1038/s41523-021-00337-2 PMC8492731

[14] Mu, L. et al. Favorable outcome associated with an IGF-1 ligand signature in breast cancer. *Breast Cancer Res. Treat.* **133**, 321–331 (2012). 22297468 10.1007/s10549-012-1952-5

[15] Creighton, C. J. et al. Insulin-like growth factor-I activates gene transcription programs strongly associated with poor breast cancer prognosis. *J. Clin. Oncol.* **26**, 4078 (2008). 18757322 10.1200/JCO.2007.13.4429 PMC2654368

[16] Du, L. et al. Predicted sensitivity to endocrine therapy for stage II-III hormone receptor-positive and HER2-negative (HR+/HER2−) breast cancer before chemo-endocrine therapy. *Ann. Oncol.* **32**, 642–651 (2021). 33617937 10.1016/j.annonc.2021.02.011 PMC12236464

[17] Pusztai, L. et al. Durvalumab with olaparib and paclitaxel for high-risk HER2-negative stage II/III breast cancer: results from the adaptively randomized I-SPY2 trial. *Cancer Cell* **39**, 989–998.e985 (2021). 34143979 10.1016/j.ccell.2021.05.009 PMC11064785

[18] Nanda, R. et al. Effect of pembrolizumab plus neoadjuvant chemotherapy on pathologic complete response in women with early-stage breast cancer: an analysis of the ongoing phase 2 adaptively randomized I-SPY2 trial. *JAMA Oncol.* **6**, 676–684 (2020). 32053137 10.1001/jamaoncol.2019.6650 PMC7058271

[19] Wulfkuhle, J. D. et al. Evaluation of the HER/PI3K/AKT family signaling network as a predictive biomarker of pathologic complete response for patients with breast cancer treated with neratinib in the I-SPY 2 Trial. *JCO Precis. Oncol.* **2**, 18 (2018). 10.1200/PO.18.00024 PMC7446527 32914002

[20] Wolf, D. M. et al. Redefining breast cancer subtypes to guide treatment prioritization and maximize response: predictive biomarkers across 10 cancer therapies. *Cancer Cell* **40**, 609–623.e606 (2022). 35623341 10.1016/j.ccell.2022.05.005 PMC9426306

[21] Magbanua, M. J. M. et al. Clinical significance and biology of circulating tumor DNA in high-risk early-stage HER2-negative breast cancer receiving neoadjuvant chemotherapy. *Cancer Cell* **41**, 1091–1102.e1094 (2023). 37146605 10.1016/j.ccell.2023.04.008 PMC10330514

[22] Park, J. W. et al. Adaptive randomization of neratinib in early breast cancer. *N. Engl. J. Med.* **375**, 11–22 (2016). 27406346 10.1056/NEJMoa1513750 PMC5259558

[23] Rugo, H. S. et al. Adaptive randomization of veliparib-carboplatin treatment in breast cancer. *N. Engl. J. Med.* **375**, 23–34 (2016). 27406347 10.1056/NEJMoa1513749 PMC5259561

[24] Evdokimova, V. et al. IGFBP7 binds to the IGF-1 receptor and blocks its activation by insulin-like growth factors. *Sci. Signal* **5**, ra92 (2012). 23250396 10.1126/scisignal.2003184

[25] Oh, Y. et al. Synthesis and characterization of insulin-like growth factor binding protein (IGFBP)-7: recombinant human mac25 protein specifically binds IGF-I and II. *J. Biol. Chem.* **271**, 30322 (1996). 8939990 10.1074/jbc.271.48.30322

[26] Artico, L. L. et al. Physiologic IGFBP7 levels prolong IGF1R activation in acute lymphoblastic leukemia. *Blood Adv.* **5**, 3633–3646 (2021). 34438446 10.1182/bloodadvances.2020003627 PMC8945593

[27] Artico, L. L. et al. IGFBP7 fuels the glycolytic metabolism in B-cell precursor acute lymphoblastic leukemia by sustaining activation of the IGF1R–Akt–GLUT1 axis. *Int. J. Mol. Sci.* **24**, 9679 (2023). 37298628 10.3390/ijms24119679 PMC10253689

[28] Godina, C. et al. Prognostic impact of tumor-specific insulin-like growth factor binding protein 7 (IGFBP7) levels in breast cancer: a prospective cohort study. *Carcinogenesis* **42**, 1314–1325 (2021). 34606580 10.1093/carcin/bgab090 PMC8598394

[29] Rosendahl, A. H. et al. Pre- and postoperative circulating IGF-I, IGFBP-3, and IGFBP-7 levels in relation to endocrine treatment and breast cancer recurrence: a nested case-control study. *Front. Oncol.* **11**, 626058 (2021). 33767994 10.3389/fonc.2021.626058 PMC7986849

[30] Luca, B. A. et al. Atlas of clinically distinct cell states and ecosystems across human solid tumors. *Cell* **184**, 5482–5496.e5428 (2021). 34597583 10.1016/j.cell.2021.09.014 PMC8526411

[31] Harris, B. H. et al. Obesity: a perfect storm for carcinogenesis. *Cancer Metastasis Rev.* **41**, 491–515 (2022). 36038791 10.1007/s10555-022-10046-2 PMC9470699

[32] Kang, C., LeRoith, D. & Gallagher, E. J. Diabetes, obesity, and breast cancer. *Endocrinology* **159**, 3801–3812 (2018). 30215698 10.1210/en.2018-00574 PMC6202853

[33] Akshintala, S. et al. Phase I trial of ganitumab plus dasatinib to cotarget the insulin-like growth factor 1 receptor and Src family kinase YES in Rhabdomyosarcoma. *Clin. Cancer Res.* **29**, 3329–3339 (2023). 37398992 10.1158/1078-0432.CCR-23-0709 PMC10529967

[34] Lu, Y., Zi, X., Zhao, Y., Mascarenhas, D. & Pollak, M. Insulin-like growth factor-I receptor signaling and resistance to trastuzumab (Herceptin). *J. Natl Cancer Inst.* **93**, 1852–1857 (2001). 11752009 10.1093/jnci/93.24.1852

[35] Schmid, P. et al. Event-free survival with pembrolizumab in early triple-negative breast cancer. *N. Engl. J. Med.* **386**, 556–567 (2022). 35139274 10.1056/NEJMoa2112651

[36] Bianchini, G., De Angelis, C., Licata, L. & Gianni, L. Treatment landscape of triple-negative breast cancer—expanded options, evolving needs. *Nat. Rev. Clin. Oncol.* **19**, 91–113 (2022). 34754128 10.1038/s41571-021-00565-2

[37] Davison, Z., de Blacquière, G. E., Westley, B. R. & May, F. E. Insulin-like growth factor-dependent proliferation and survival of triple-negative breast cancer cells: implications for therapy. *Neoplasia* **13**, 504–515 (2011). 21677874 10.1593/neo.101590 PMC3114244

[38] Hopkins, B. D. et al. Suppression of insulin feedback enhances the efficacy of PI3K inhibitors. *Nature* **560**, 499–503 (2018). 30051890 10.1038/s41586-018-0343-4 PMC6197057

[39] Paddock, M. N., Field, S. J. & Cantley, L. C. Treating cancer with phosphatidylinositol-3-kinase inhibitors: increasing efficacy and overcoming resistance. *J. Lipid Res.* **60**, 747–752 (2019). 30718284 10.1194/jlr.S092130 PMC6446698

[40] Pollak, M. Diet boosts the effectiveness of a cancer drug. *Nature* **560**, 439–440 (2018). 30127476 10.1038/d41586-018-05871-x

[41] Vaduganathan, M. et al. Stress cardiac biomarkers, cardiovascular and renal outcomes, and response to canagliflozin. *J. Am. Coll. Cardiol.* **79**, 432–444 (2022). 35115099 10.1016/S0735-1097(22)01423-1 PMC8972403

[42] Saal, L. H. et al. The Sweden Cancerome Analysis Network—Breast (SCAN-B) Initiative: a large-scale multicenter infrastructure towards implementation of breast cancer genomic analyses in the clinical routine. *Genome Med.* **7**, 20 (2015). 25722745 10.1186/s13073-015-0131-9 PMC4341872

[43] Rydén, L. et al. Minimizing inequality in access to precision medicine in breast cancer by real-time population-based molecular analysis in the SCAN-B initiative. *Br. J. Surg.* **105**, e158–e168 (2018). 29341157 10.1002/bjs.10741 PMC5817401

[44] Staaf, J. et al. RNA sequencing-based single sample predictors of molecular subtype and risk of recurrence for clinical assessment of early-stage breast cancer. *NPJ Breast Cancer* **8**, 94 (2022). 35974007 10.1038/s41523-022-00465-3 PMC9381586

[45] Yee, D. et al. Association of event-free and distant recurrence-free survival with individual-level pathologic complete response in neoadjuvant treatment of stages 2 and 3 breast cancer: three-year follow-up analysis for the I-SPY2 adaptively randomized clinical trial. *JAMA Oncol.* **6**, 1355–1362 (2020). 32701140 10.1001/jamaoncol.2020.2535 PMC7378873

[46] Goodwin, P. J. et al. Effect of metformin vs. placebo on invasive disease-free survival in patients with breast cancer: the MA.32 randomized clinical trial. *JAMA* **327**, 1963–1973 (2022). 35608580 10.1001/jama.2022.6147 PMC9131745

[47] Fredlund, E. et al. The gene expression landscape of breast cancer is shaped by tumor protein p53 status and epithelial-mesenchymal transition. *Breast Cancer Res.* **14**, R113 (2012). 22839103 10.1186/bcr3236 PMC3680939

[48] Ritchie, M. E. et al. limma powers differential expression analyses for RNA-sequencing and microarray studies. *Nucleic Acids Res.* **43**, e47 (2015). 25605792 10.1093/nar/gkv007 PMC4402510

[49] Wu, T. et al. clusterProfiler 4.0: a universal enrichment tool for interpreting omics data. *Innovation (Cambridge)* **2**, 100141 (2021). 10.1016/j.xinn.2021.100141 PMC8454663 34557778

[50] Liberzon, A. et al. The Molecular Signatures Database (MSigDB) hallmark gene set collection. *Cell Syst.* **1**, 417–425 (2015). 26771021 10.1016/j.cels.2015.12.004 PMC4707969

[51] McShane, L. M. et al. Reporting recommendations for tumor marker prognostic studies (REMARK). *J. Natl Cancer Inst.* **97**, 1180–1184 (2005). 16106022 10.1093/jnci/dji237

