# Supplementary material for: Targeting IGF-IR improves neoadjuvant chemotherapy efficacy in breast cancers with low IGFBP7 expression
Source: NPJ Precis Oncol. 2024 Oct 3;8:212. doi: 10.1038/s41698-024-00712-9 (PMC11450189; doi:10.1038/s41698-024-00712-9)
Supplement: Supplementary file 1 — Supplementary Figures and Tables [file 41698_2024_712_MOESM1_ESM.pdf]

Low tumor IGFBP7 expression predicts complete pathological response of breast cancer patients to IGF-1R targeting with ganitumab

Christopher Godina[1], Michael N Pollak[2], Helena Jernström[1].

1 Division of Oncology, Department of Clinical Sciences in Lund, Lund University and Skåne University Hospital, Barngatan 4, SE-221 85 Lund, Sweden

2 Lady Davis Institute for Medical Research, Jewish General Hospital and Department of Oncology, McGill University, Montreal, QC, Canada

Table of Contents

Supplementary Figure Legends……………………………………………………………1

Supplementary Figure 1…………………………………………………………………2

Supplementary Figure 2…………………………………………………………………3

Supplementary Table 1……………………………………………………………………4

Supplementary Table 2……………………………………………………………………5

Supplementary Table 3……………………………………………………………………6

Supplementary Table 4……………………………………………………………………7

Supplementary Table 5……………………………………………………………………8

Supplementary Table 6-13 is available separately as an .xlsx file (Additional File 1).

Supplementary figure legends

Supplementary Fig. 1. Flowchart of included and excluded patients in SCAN-B

Supplementary Fig. 2. *IGFBP7* expression in relation to molecular features

Correlation plots of Pearson correlations coefficients of *IGFBP7* gene expression and 15 genes in the IGF/Insulin pathway (*INS, INSR, IRS1, IRS2, IGF1, IGF2, IGFALS, IGF1R, IGF2R, IGFBP1, IGFBP2, IGFBP3, IGFBP4, IGFBP5,* and *IGFBP6*) in (**A**) I-SPY2, and (**B**) SCAN-B. Red indicates positive correlation, blue indicates negative correlation, and grey indicates no correlation. Bar plots of Pearson correlations coefficients of *IGFBP7* gene expression (continuous) and the eight gene modules (Stroma, Lipid, Immune Response, Mitotic Checkpoint, Mitotic Progression, Basal, Early Response, Steroid Response) in (**C**) I-SPY2, and (**D**) SCAN-B. The eight gene modules are presented on the x-axis and the Pearson correlation values are presented on the y-axis. *IGFBP7* expression as a continuous variable by PAM50 subtype in (**E**) I-SPY2 and (**F**) SCAN-B. SCAN-B. *IGFBP7* expression as a continuous variable by receptor subtype in (**G**) I-SPY2 and (**H**) SCAN-B. Violin plots illustrate the distribution of *IGFBP7* expression by PAM50 subtype and receptor subtype with overlaying box plots. In the box plots, the boundary of the box closest to zero indicates the 25th percentile, a black line within the box marks the median, and the boundary of the box farthest from zero indicates the 75th

percentile. Points above (Q3 + 1.5*interquartile range (IQR)) and below the (Q1 − 1.5*interquartile range (IQR)) the whiskers indicate outliers. Fig panels E-H are presented and the Kruskal-Wallis test was used for statistical analysis.

Supplementary Fig. 3. *IGFBP7* gene expression in relation to different immune signatures

Correlations of *IGFBP7* gene expression and different immune signatures were depicted using correlation plots from expression correlation matrices of gene signatures and genes. The correlation plot corresponds to Pearson correlation coefficients calculated across the entire biomarker study populations in (**A**) I-SPY2 and (**B**) SCAN-B. Red indicates positive correlations, blue indicates negative correlations, and grey indicates no correlation. Each correlation plot is clustered using hierarchical clustering and the order of signatures are determined by the clustering.

Supplementary Fig. 4. Hallmark signature analysis

Dot plots showing activated and suppressed Hallmark Signatures from gene set enrichment analysis (GSEA; Hallmark signatures) of genes ranked by log2FC and *P*-values in *IGFBP7* Q4 compared to *IGFBP7* Q1 tumors in (**A**) I-SPY2, and (**B**) SCAN-B**.** The Hallmark Signatures gene sets are ranked by gene ratio (x-axis), which is the percentage of significant genes over the total number of genes in a given pathway. The dots size indicates the total number of genes in a given gene set (Hallmark Signature). Red indicates higher and blue lower FDR-adjusted *P*-values from GSEA. However, all FDR-adjusted *P*-values for the shown signatures are significant ($P < 0.05$). A Venn diagram (**C**) of Hallmarks signatures found to be activated or suppressed in both cohorts. A Circos diagram (**D**) shows the relationship between the leading-edge genes of the hallmarks, EMT, angiogenesis, coagulation, JAK2/STAT5 signaling, and TGF-β signaling with the highest Jaccard index. Concentrically from the outside, the number of genes is plotted for leading subsets of the five most similar Hallmarks that are the same in both I-SPY2 and SCAN-B. The arcs in the center of the circus diagram indicate the number of leading-edge subset genes that are shared between each hallmark.

Supplementary Fig. 1.

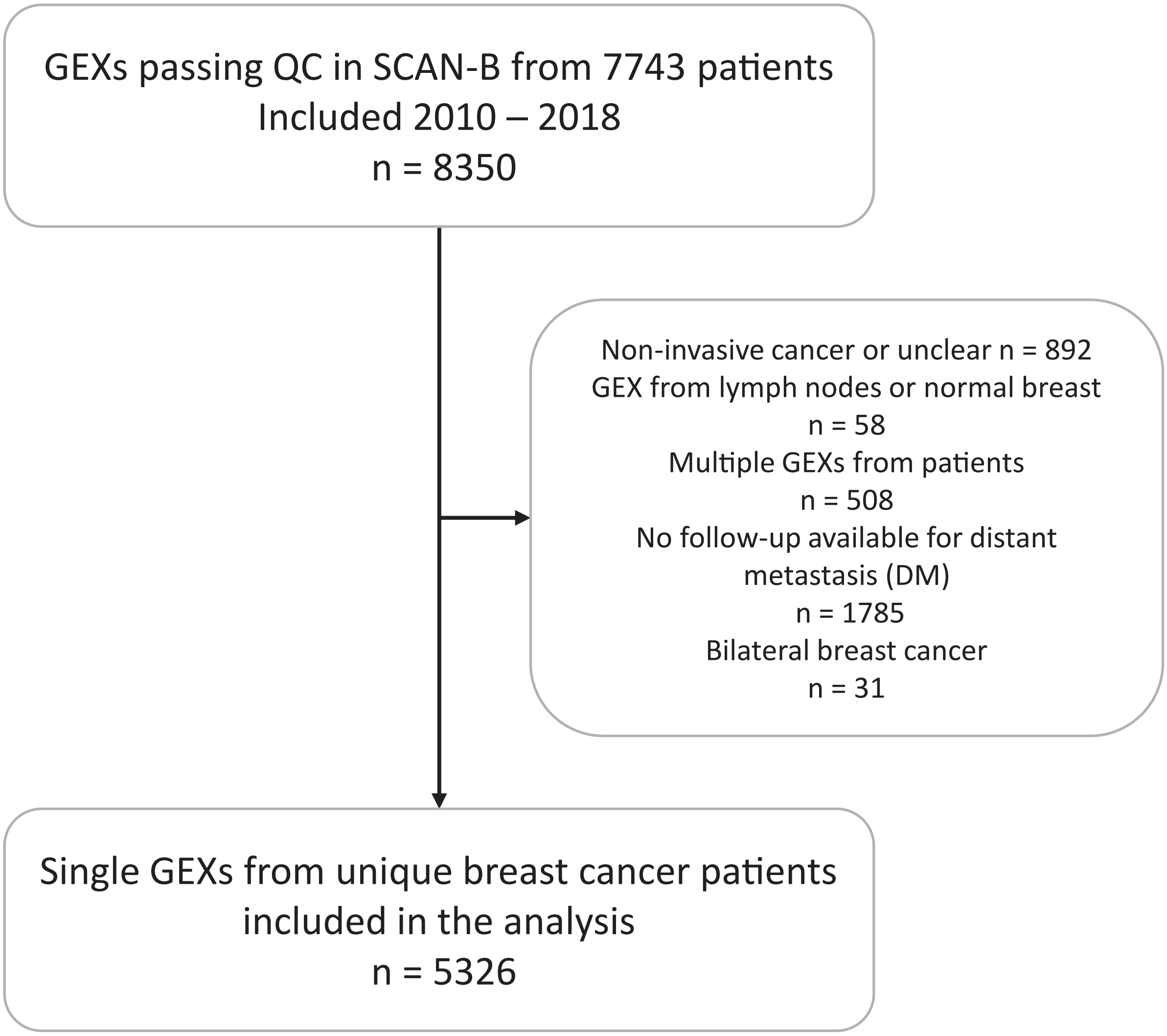

Supplementary Fig. 2.

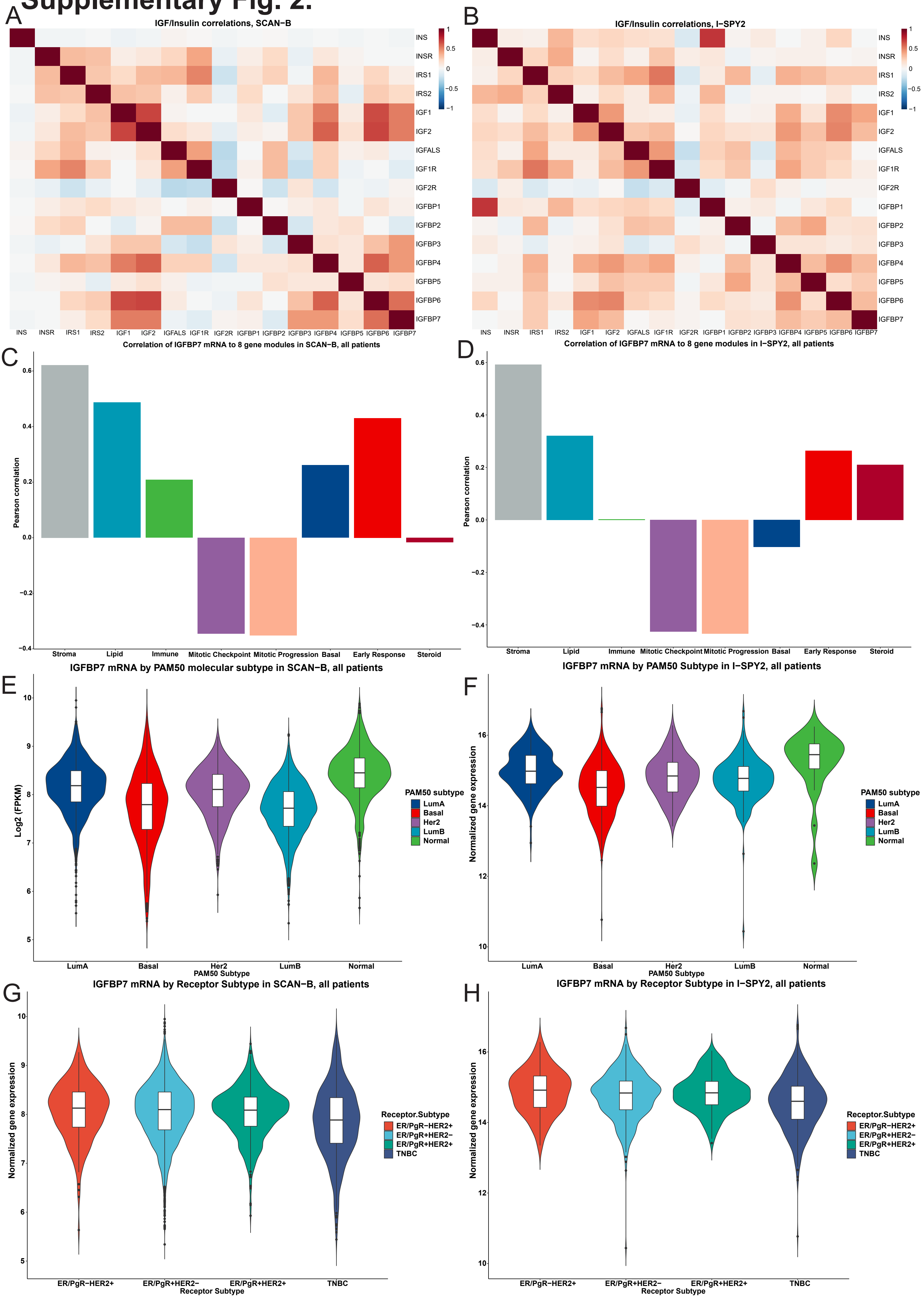

Supplementary Fig. 3.

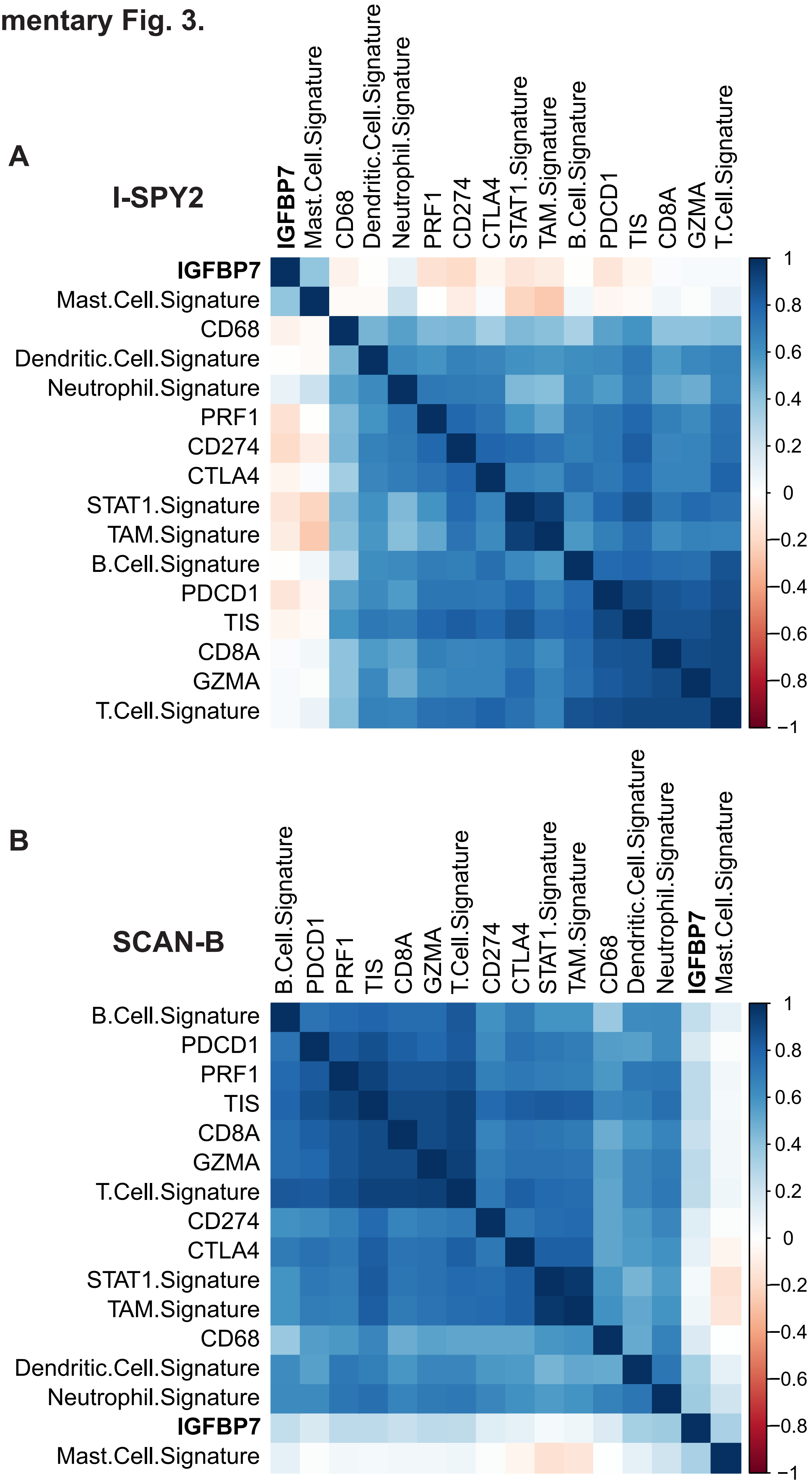

Supplementary Fig. 4.

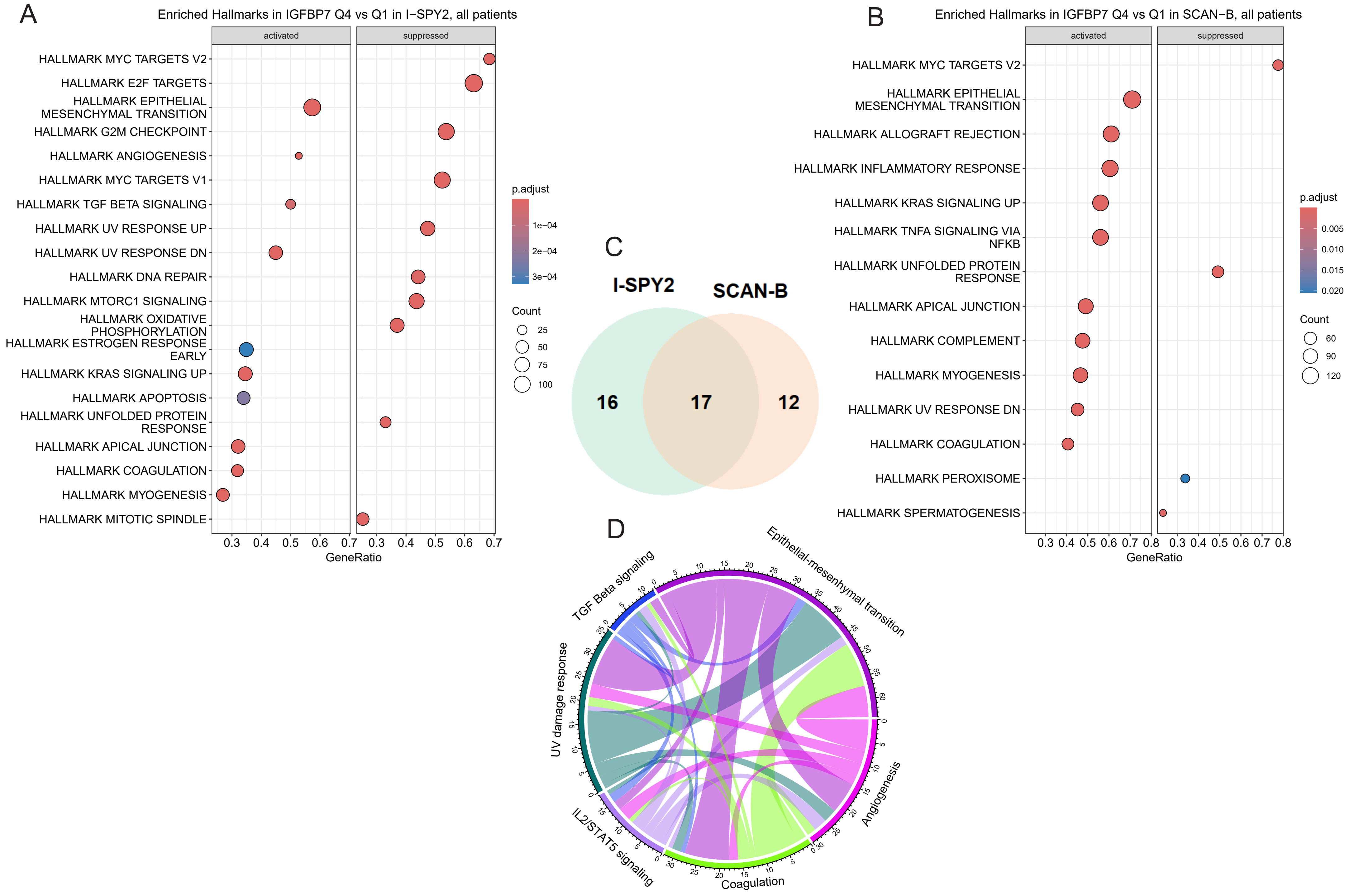

Supplementary Table 1 Odds ratio of achieving a pCR in relation *IGFBP7* gene expression, all patients (arms) in I-SPY2

	IGFBP7 continuous		IGFBP7 quartiles	
**Variable**	**OR**[1]	**95% CI**[1]	**OR**[1]	**95% CI**[1]
**IGFBP7 Continuous**	0.99	0.79, 1.25		
**IGFBP7 Quartiles**				
Q1			Ref.	
Q2			0.77	0.50, 1.20
Q3			1.19	0.76, 1.85
Q4			0.96	0.60, 1.52
**ER/PgR+**	0.72	0.46, 1.10	0.71	0.46, 1.09
**HER2+**	3.32	1.88, 5.85	3.34	1.90, 5.90
**MP2**	1.54	0.97, 2.46	1.60	1.00, 2.56
**Immune+**	2.89	2.03, 4.15	2.88	2.02, 4.14
**DRD+**	1.64	1.03, 2.65	1.69	1.05, 2.73
**PAM50 Subtype**				
LumA	Ref.		Ref.	
Basal	1.86	0.83, 4.21	1.84	0.82, 4.17
Her2	3.32	1.70, 6.60	3.34	1.71, 6.65
LumB	1.58	0.86, 2.95	1.65	0.90, 3.09
Normal	2.00	0.59, 6.43	2.02	0.60, 6.50
**Trial Arm**				
Chemotherapy-alone	Ref.		Ref.	
AMG386	2.10	1.19, 3.73	2.15	1.22, 3.82
Ganetespib	1.74	0.91, 3.28	1.69	0.89, 3.20
Ganitumab + metformin	1.76	0.92, 3.32	1.75	0.92, 3.30
MK2206	1.89	1.01, 3.54	1.85	0.99, 3.47
Neratinib	1.98	1.08, 3.64	2.01	1.10, 3.70
Pembrolizumab	5.38	2.70, 10.8	5.30	2.66, 10.7
Pertuzumab	4.91	2.09, 11.8	5.21	2.20, 12.6
Trastuzumab-emtansine	3.82	1.65, 8.99	3.94	1.69, 9.32
Carboplatin + ABT888	3.55	1.80, 7.04	3.56	1.80, 7.06

[1]OR = Odds Ratio, CI = Confidence Interval

Control: Paclitaxel (+Trastuzumab if HER2+) followed by doxorubicin and cyclophosphamide

Supplementary Table 2. Descriptive statistics of *IGFBP7* quartiles in relation to clinicopathological characteristics in I-SPY2

			IGFBP7 Quartiles			
	All patients[1]	Missing	**Q1**, n = 244[1]	**Q2**, n = 244[1]	**Q3**, n = 243[1]	**Q4**, n = 243[1]
**ER/PgR+**	528 (54%)	0	103 (42%)	133 (55%)	153 (63%)	139 (57%)
**HER2+**	244 (25%)	0	35 (14%)	71 (29%)	65 (27%)	73 (30%)
**MP2**	476 (49%)	0	168 (69%)	128 (52%)	97 (40%)	83 (34%)
**Immune+**	446 (46%)	0	140 (57%)	103 (42%)	101 (42%)	102 (42%)
**DRD+**	364 (37%)	3	128 (52%)	94 (39%)	71 (29%)	71 (29%)
**PAM50 Subtype**		15				
LumA	172 (18%)		16 (6.6%)	36 (15%)	57 (24%)	63 (26%)
Basal	406 (42%)		154 (64%)	100 (42%)	82 (34%)	70 (29%)
Her2	140 (15%)		27 (11%)	35 (15%)	34 (14%)	44 (18%)
LumB	218 (23%)		42 (17%)	66 (28%)	63 (26%)	47 (20%)
Normal	23 (2.4%)		2 (0.8%)	2 (0.8%)	3 (1.3%)	16 (6.7%)
**pCR**	313 (32%)	0	86 (35%)	74 (30%)	79 (33%)	74 (30%)
**Trial Arm**		0				
Chemotherapy-alone	205 (21%)		55 (23%)	45 (18%)	53 (22%)	52 (21%)
AMG386	133 (14%)		30 (12%)	37 (15%)	27 (11%)	39 (16%)
Ganetespib	93 (9.5%)		28 (11%)	22 (9.0%)	27 (11%)	16 (6.6%)
Ganitumab + metformin	105 (11%)		32 (13%)	29 (12%)	26 (11%)	18 (7.4%)
MK2206	94 (9.7%)		24 (9.8%)	17 (7.0%)	26 (11%)	27 (11%)
Neratinib	112 (11%)		24 (9.8%)	32 (13%)	28 (12%)	28 (12%)
Pembrolizumab	69 (7.1%)		8 (3.3%)	11 (4.5%)	16 (6.6%)	34 (14%)
Pertuzumab	44 (4.5%)		7 (2.9%)	19 (7.8%)	10 (4.1%)	8 (3.3%)
Trastuzumab-emtansine	52 (5.3%)		7 (2.9%)	17 (7.0%)	15 (6.2%)	13 (5.3%)
Carboplatin + ABT888	67 (6.9%)		29 (12%)	15 (6.1%)	15 (6.2%)	8 (3.3%)

[1]n (%)

Control: Paclitaxel (+Trastuzumab if HER2+) followed by Anthracyclines

Supplementary Table 3. Descriptive statistics of IGFBP7 quartiles in relation to clinicopathological characteristics in SCAN-B

			IGFBP7 Quartiles			
	All patients[1]	Missing	**Q1**, n = 1,332[1]	**Q2**, n = 1,332[1]	**Q3**, n = 1,331[1]	**Q4**, N = 1,331[1]
**Age at diagnosis, years**		0				
-40	270 (5.1%)		72 (5.4%)	51 (3.8%)	85 (6.4%)	62 (4.7%)
41-50	874 (16%)		176 (13%)	235 (18%)	260 (20%)	203 (15%)
51-60	1,045 (20%)		233 (17%)	253 (19%)	282 (21%)	277 (21%)
61-70	1,661 (31%)		396 (30%)	426 (32%)	403 (30%)	436 (33%)
71-80	995 (19%)		279 (21%)	249 (19%)	211 (16%)	256 (19%)
81-	481 (9.0%)		176 (13%)	118 (8.9%)	90 (6.8%)	97 (7.3%)
**Invasive tumor size** (pT2/3/4)	1,783 (35%)	166	530 (41%)	421 (32%)	403 (31%)	429 (33%)
**Lymph node status** (pN1/2/3)	1,873 (37%)	213	461 (36%)	475 (37%)	497 (39%)	440 (34%)
**Histological type**		37				
Ductal	4,182 (79%)		1,083 (82%)	1,085 (82%)	1,083 (82%)	931 (71%)
Lobular	732 (14%)		108 (8.2%)	137 (10%)	167 (13%)	320 (24%)
Other or Mixed	375 (7.1%)		131 (9.9%)	100 (7.6%)	77 (5.8%)	67 (5.1%)
**Histological grade**		382				
I	791 (16%)		130 (11%)	181 (15%)	224 (18%)	256 (21%)
II	2,443 (49%)		512 (42%)	621 (50%)	626 (50%)	684 (55%)
III	1,710 (35%)		588 (48%)	437 (35%)	391 (32%)	294 (24%)
**ER+**	4,497 (85%)	49	1,047 (79%)	1,138 (87%)	1,168 (88%)	1,144 (87%)
**PgR+**	3,725 (71%)	51	871 (66%)	965 (73%)	959 (72%)	930 (71%)
**HER2+**	702 (14%)	149	145 (11%)	192 (15%)	213 (16%)	152 (12%)
**TNBC**	525 (10%)	278	208 (17%)	117 (9.3%)	91 (7.2%)	109 (8.6%)
**Endocrine therapy**	4,109 (78%)	85	965 (74%)	1,046 (79%)	1,053 (81%)	1,045 (80%)
**Chemotherapy**	2,243 (43%)	85	616 (47%)	583 (44%)	565 (43%)	479 (36%)
**Trastuzumab**	585 (11%)	85	118 (9.1%)	165 (13%)	172 (13%)	130 (9.9%)
**PAM50 Subtype**		0				
LumA	2,255 (42%)		365 (27%)	576 (43%)	650 (49%)	664 (50%)
Basal	471 (8.8%)		204 (15%)	114 (8.6%)	72 (5.4%)	81 (6.1%)
Her2	641 (12%)		131 (9.8%)	167 (13%)	193 (15%)	150 (11%)
LumB	1,275 (24%)		574 (43%)	391 (29%)	224 (17%)	86 (6.5%)
Normal	684 (13%)		58 (4.4%)	84 (6.3%)	192 (14%)	350 (26%)
**PAM50 ROR**		292				
High	2,361 (47%)		824 (66%)	674 (54%)	507 (40%)	356 (28%)
Intermediate	738 (15%)		205 (16%)	185 (15%)	204 (16%)	144 (11%)
Low	1,935 (38%)		219 (18%)	399 (32%)	554 (44%)	763 (60%)

[1]n (%)

Supplementary Table 4. *IGFBP7* modeled in quartiles gene expression in relation to clinical outcome in SCAN-B

	Recurrence-free interval		Distant metastasis-free interval	
**Variables**	**HR**[1]	**95% CI**[1]	**HR**[1]	**95% CI**[1]
**Crude**				
**IGFBP7 quartiles**				
Q1	Ref.		Ref.	
Q2	0.95	0.75, 1,21	0.99	0.76, 1,31
Q3	0.92	0.72, 1.16	0.93	0.71, 1.23
Q4	0.96	0.76, 1.22	1.00	0.77, 1.32
**Multivariable**				
**IGFBP7 quartiles**				
Q1	Ref.		Ref.	
Q2	1.13	0.87, 1.47	1.26	0.93, 1.71
Q3	1.20	0.91, 1.58	1.37	1.00, 1.89
Q4	1.37	1.04, 1.82	1.60	1.15, 2.22
**Age** (5-year bin)	1.00	0.99, 1.01	1.01	1.00, 1.02
**Tumor size** (pT2/3/4)	1.90	1.55, 2.32	2.26	1.78, 2.85
**Lymph node status** (pN1/2/3)	1.31	1.06, 1.62	1.57	1.24, 2.01
**Grade III**	1.27	0.98, 1.63	1.29	0.97, 1.73
**ER+**	1.52	0.94, 2.46	1.06	0.59, 1.91
**PgR+**	0.90	0.69, 1.17	0.81	0.60, 1.09
**HER2+**	1.95	1.27, 2.99	2.09	1.32, 3.30
**PAM50 ROR High**	2.20	1.61, 3.01	2.47	1.71, 3.58
**PAM50 subtype**				
LumA	Ref.		Ref.	
Basal	1.53	0.91, 2.58	1.68	0.93, 3.04
Her2	1.60	1.06, 2.42	1.58	0.99, 2.53
LumB	1.21	0.87, 1.70	1.29	0.87, 1.90
Normal	1.40	0.99, 1.97	1.47	0.97, 2.23
**Chemotherapy**	0.77	0.59, 1.00	0.91	0.67, 1.24
**Endocrine therapy**	0.45	0.32, 0.63	0.75	0.46, 1.21
**Trastuzumab**	0.30	0.18, 0.51	0.29	0.16, 0.51

Supplementary Table 5. *IGFBP7* gene expression modeled as a continuous variable in relation to clinical outcome in SCAN-B

	Recurrence-free interval		Distant metastasis-free interval	
**Variables**	**HR**[1]	**95% CI**[1]	**HR**[1]	**95% CI**[1]
**Crude**				
**IGFBP7 continuous**	1.01	0.88, 1.16	1.02	0.87, 1.20
**Multivariable**				
**IGFBP7 continuous**	1.29	1.09, 1.53	1.41	1.16, 1.73
**Age** (5-year bin)	1.00	0.99, 1.01	1.01	1.00, 1.02
**Tumor size** (pT2/3/4)	1.92	1.57, 2.34	2.27	1.80, 2.87
**Lymph node status** (pN1/2/3)	1.30	1.05, 1.60	1.56	1.23, 1.99
**Grade III**	1.27	0.99, 1.63	1.29	0.97, 1.73
**ER+**	1.52	0.94, 2.46	1.05	0.58, 1.91
**PgR+**	0.90	0.69, 1.18	0.81	0.61, 1.09
**HER2+**	1.92	1.25, 2.93	2.07	1.31, 3.26
**PAM50 ROR High**	2.23	1.63, 3.05	2.52	1.74, 3.64
**PAM50 subtype**				
LumA	Ref.		Ref.	
Basal	1.57	0.93, 2.65	1.72	0.95, 3.11
Her2	1.61	1.07, 2.43	1.59	0.99, 2.55
LumB	1.24	0.89, 1.73	1.31	0.89, 1.93
Normal	1.37	0.97, 1.93	1.43	0.94, 2.17
**Chemotherapy**	0.77	0.59, 1.00	0.91	0.67, 1.24
**Endocrine therapy**	0.45	0.32, 0.63	0.75	0.46, 1.21
**Trastuzumab**	0.31	0.18, 0.52	0.29	0.17, 0.52
